# Presence of Cartilage Stem/Progenitor Cells in Adult Mice Auricular Perichondrium

**DOI:** 10.1371/journal.pone.0026393

**Published:** 2011-10-19

**Authors:** Shinji Kobayashi, Takanori Takebe, Yun-Wen Zheng, Mitsuru Mizuno, Yuichiro Yabuki, Jiro Maegawa, Hideki Taniguchi

**Affiliations:** 1 Department of Regenerative Medicine, Yokohama City University Graduate School of Medicine, Kanazawa-ku, Yokohama, Kanagawa, Japan; 2 Department of Plastic and Reconstructive Surgery, Yokohama City University Graduate School of Medicine, Kanazawa-ku, Yokohama, Kanagawa, Japan; 3 Advanced Medical Research Center, Yokohama City University, Yokohama, Kanagawa, Japan; 4 Department of Plastic and Reconstructive Surgery, Kanagawa Children's Medical Center, Minami-ku, Yokohama, Japan; Harvard Medical School, United States of America

## Abstract

**Background:**

Based on evidence from several other tissues, cartilage stem/progenitor cells in the auricular cartilage presumably contribute to tissue development or homeostasis of the auricle. However, no definitive studies have identified or characterized a stem/progenitor population in mice auricle.

**Methodology/Principal Findings:**

The 5-bromo-2′-deoxyuridine (BrdU) label-retaining technique was used to label dividing cells in fetal mice. Observations one year following the labeling revealed that label-retaining cells (LRCs) were present specifically in auricular perichondrium at a rate of 0.08±0.06%, but LRCs were not present in chondrium. Furthermore, LRCs were successfully isolated and cultivated from auricular cartilage. Immunocytochemical analyses showed that LRCs express CD44 and integrin-α_5_. These LRCs, putative stem/progenitor cells, possess clonogenicity and chondrogenic capability *in vitro*.

**Conclusions/Significance:**

We have identified a population of putative cartilage stem/progenitor cells in the auricular perichondrium of mice. Further characterization and utilization of the cell population should improve our understanding of basic cartilage biology and lead to advances in cartilage tissue engineering and novel therapeutic strategies for patients with craniofacial defects, including long-term tissue restoration.

## Introduction

Evidence from several tissues, including epidermis, cornea, adipose and skeletal muscle indicate that stem cells support tissue maintenance by balancing self-renewal and differentiation [1], [2], [3], [4]. Auricular cartilage, known as elastic cartilage, also is considered that stem/progenitor cells support tissue maintenance after birth. In regard to the other type of cartilage maintenance, costal cartilage known as hyaline cartilage is clinically regenerated from surrounding perichondrium, suggesting the presence of stem/progenitor cells in perichondrium [5], [6], [7]. Although several in vitro studies indicate that the auricular perichondrium can support cartilage regeneration and that the auricular perichondrium, like that surrounding the costal cartilage, harbors multipotent progenitor cells, no definitive studies have demonstrated the existence of stem/progenitor cells in the auricle cartilage or surrounding perichondrium [8].

Identification of cartilage stem/progenitor cells in the auricle is useful for understanding the development of the auricle system and potentially for developing a source of cells for cartilage tissue engineering. Conventional treatments for patients with craniofacial defects, which involve transplantation of auto-costal cartilage, have several critical problems, including donor limitation, donor involvement, and absorption over time [7], [9], [10]. To overcome these problems, alternative treatments that are based on elastic cartilage engineering and that use terminally differentiated chondrocytes derived from the auricle have been developed [11]. However, using differentiated chondrocytes puts a significant burden on donor sites; moreover, these grafts, which do not contain self-renewing stem cells, are absorbed over time not maintained. These clinical limitations may be overcome by the use of stem cells, as self-renewing stem cells can lead to permanent restoration of tissues characterized by high and continuous self-renewal [12].

The aim of our study was to determine whether stem/progenitor cells were present in auricular cartilage of adult mice. Initially, we performed a label-retention assay, which has been used to identify putative stem cells in several tissue types, in the developing auricle of mice [13], [14], [15]. Furthermore, we successfully isolated and cultivated a population of cells that retained the label, designated, long-term label retaining cells (LRCs), and characterized these LRCs based on expression of cell-surface markers.

## Results

### Changes in the surface area of the external ear and in the thickness of the auricular cartilage

**Figure 1:**
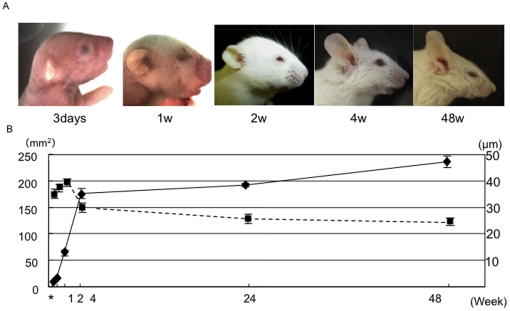
Development of murine external ears. (A) The external ear at several developmental stages. From the left, ears were photographed 3 days, 1 week, 2 weeks, 4 weeks, and 48 weeks after birth. (B) Mean surface area of the external ear and mean thickness of auricular cartilages changed during postnatal development. * ; 3 days, ; mean surface area of the external ear (mm2), ; mean thickness of auricular cartilage in the middle area of the external ear (µm) (N = 6).

The mean surface area of the outer ear was 9.4±0.7 mm^2^, 15.9±0.3 mm^2^, 63.0±5.3 mm^2^, 175.8±10.0 mm^2^, 191.6±3.3 mm^2^, and 235.9±11.1 mm^2^ (N = 6) 3 days, 1 week, 2 weeks, 4 weeks, 24 weeks, and 48 weeks, respectively, following birth (Figure 1A and B). Growth of the auricle was rapid during the 4 weeks following birth, but growth slowed after that point. Mean thickness, which was measured at the identical time points, was 32.0±2.6 mm, 37.0±2.0 mm, 38.7±1.5 mm, 29.7±1.4 mm, 24.7±1.5 mm, and 23.7±1.3 mm, respectively (N = 6) (Figure 1B). The surface area of the auricular cartilage increased throughout the first 4 postnatal weeks, while the thickness of the auricular cartilage decreased over the first 2 weeks. These results indicate that cells in the auricular cartilage, which consists of chondrium and perichondrium, were proliferating rapidly within the first 4 weeks following birth. Consequently, putative stem cells, if exist, will transition to a dormant state 4 weeks post-birth. These observations let us examine the label-retaining approach to clarify the presence of stem cells in the auricle, which was well established technique to identify the dormant or slowly cycling cells.

### Auricular perichondrium contains long-term DNA label-retaining cells (LRCs)

To identify putative stem cells in elastic cartilage of the auricle, we performed a label-retention assay that has been used to identify putative stem cells in many tissues. We performed the 5-bromo-2′-deoxyuridine (BrdU)-labeling assay on the auricle of adult mice (4 weeks old). BrdU was injected intraperitoneally, and the tissue was analyzed the following day. However, no cells in either the perichondrium or the chondrium were labeled within 24 h of the last BrdU injection (Figure S1).

**Figure 2:**
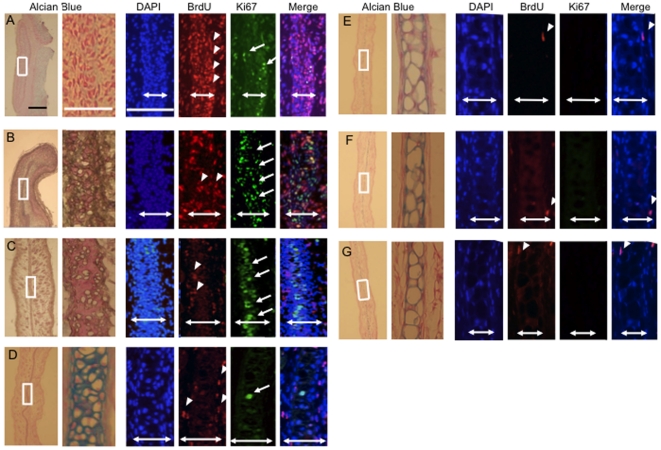
48-week chase analysis of BrdU-labeled cells and Ki67-positive cells. BrdU-labeled cells gradually decreased in auricular cartilage (White arrowheads). A few BrdU-labeled cells were present in the perichondrium of 48-week-old, BrdU-labeled offspring. No LRCs were observed in chondrocytes of the chondrium 4 weeks after BrdU labeling. No Ki67-positive cells were seen in perichondrium 1 week following birth (White arrows). No Ki67-positive cells were observed in the chondrium 2 weeks following birth. (A) 0 day, (B) 3 day, (C) 1 week, (D) 2 weeks, (E) 4 weeks, (F) 24 weeks and (G) 48 weeks. From the left, Alcian blue staining, DAPI, BrdU, Ki67, and a merged image. Two-headed arrows: the cartilage width including perichondrium. Black scale bar = 200 µm, White scale bar = 50 µm.

**Figure 3:**
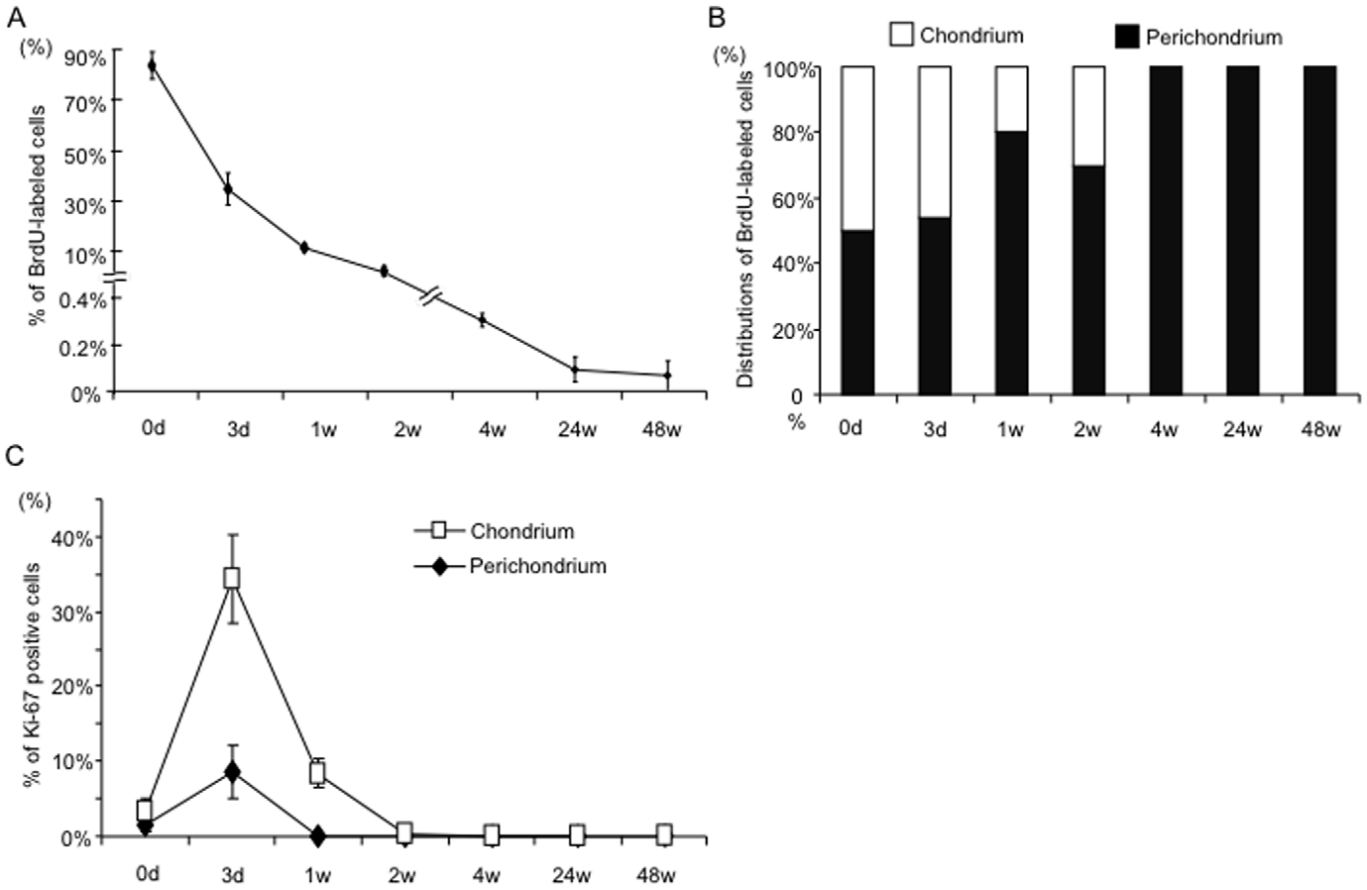
Presence of long-term label retaining cells in auricuar perichondrium 48 weeks following birth. (A) Quantification of BrdU-label retaining cells from 0 day to 48 weeks in mice auricular cartilage (N = 6). (B) The distributions of LRCs between chondrium and perichondrium layer. 4 weeks after birth, all LRCs were detected in perichondrium, but not chondrium layer. (C) Proliferation of cells in auricular cartilage. Cells in both layers extensively proliferate till 2 weeks post birth. Graph shows time course-dependent changes of Ki67-positive cell index (KI) in perichondrium and chondrium for 48 weeks after birth. ;KI of Perichondrium, ; KI of Chondrium.

Then, we treated pregnant mice with BrdU on day17 to 19 of gestation to label proliferating cells in the fetuses during auricular development. The auricles of the offspring were analyzed at multiple time points (on day 0 and day 3 and at 1, 2, 4, 24, and 48 weeks) (Figure 2A–G). To assess the efficiency of BrdU incorporation, the auricles of neonate mice were immunohistochemically labeled with monoclonal antibodies against BrdU; the majority of cells (83.6±5.4%) were labeled in neonates (N = 6) (Figure 3A). Within two weeks, the number of BrdU-positive cells, i.e., label-retaining cells (LRCs), rapidly decreased to 2.5±0.2% (N = 6). Concomitantly, localization of LRCs is restricted to the perichondrium layer (50.0%, 53.8%, 80.3% and 69.7% of BrdU positive cells, respectively)(Figure 3B). After 4 weeks, no LRCs were observed in the chondrium. However, LRCs were observed in the perichondrium even after 4 weeks (0.3±0.2% of cells) (N = 3). After 24 and 48 weeks, long-term LRCs were detected only in the perichondrium (0.1±0.05% and 0.08±0.06% of all 4,6-diamidino-2-phenylindole (DAPI)-stained cells, respectively) (N = 3, each timepoint) (Figure S2A, B, and Figure 3B). These LRCs, observed only in the perichondrium layer, seemed dormant, which is characteristic of putative stem cells.

### Transition to a dormant state after experiencing transient amplification

To determine the ratio of proliferating cells during auricular development, Ki-67 immunocytochemistry was performed. The nuclear antigen Ki-67 is a marker of proliferation and is expressed only in cycling cells. In the chondrium, Ki-67 staining was seen in 3.4±1.7%, 34±5.9%, 8.4±2.0%, and 0.4±0.8% of all DAPI-stained cells at postnatal day 0, day 3, week 1, and week 2, respectively (N = 3, each timepoint) (Figure 2A–G and 3C). After 4 weeks, no Ki-67-positive cells were observed in the chondrium. In the perichondrium, Ki-67 staining was seen in 1.5±0.8% and 8.5±3.5% of all DAPI-stained cells at postnatal day 0 and day 3 respectively, and no Ki-67-positive cells were observed at week 1 or week 2. These observations may indicate that cells in the perichondrium transitioned to the dormant state of stem cells after a transient period of amplification sometime within the first week following birth.

### LRCs localize to the opening of the external acoustic meatus

**Figure 4:**
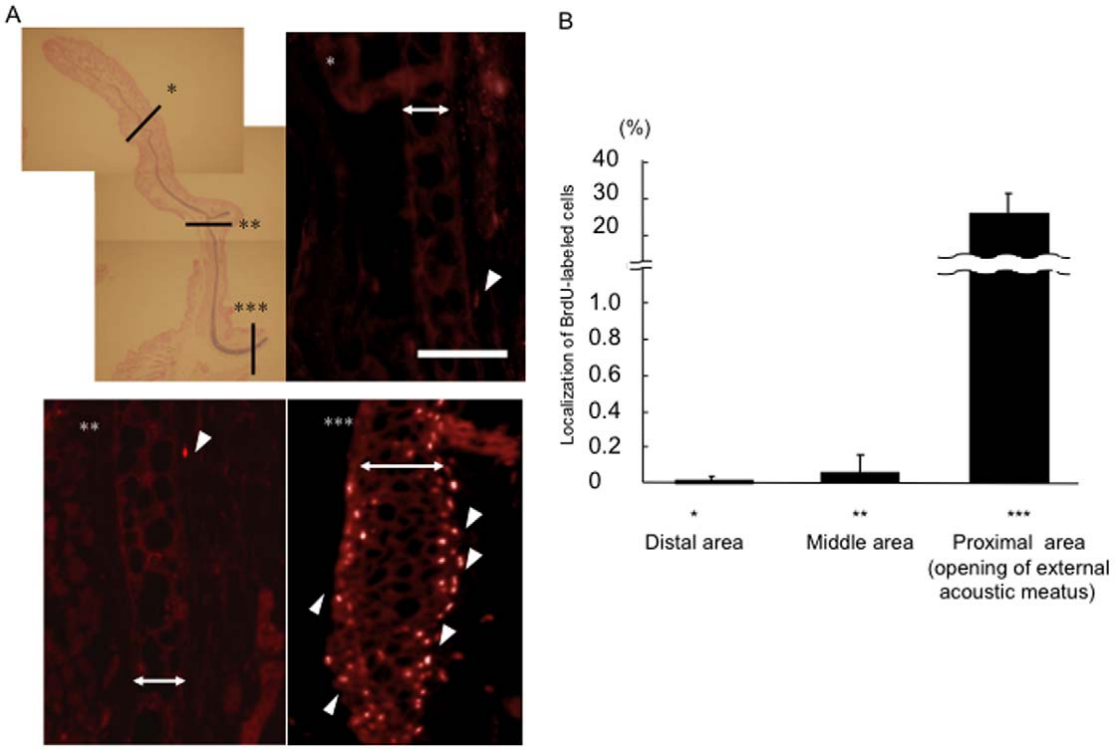
Localization of long-term LRCs in different parts of the external ear. (A) A few LRCs were present in the perichondrium in the distal and middle areas of the external ear (White arrowheads). Many LRCs were present in the opening of the external acoustic meatus (White arrowheads). Two-headed arrows: Chondrium. Scale bar = 50 µm. Original magnification: ×40 (Alcian blue staining), ×100 (BrdU staining). (B) The LRCs of the distal and middle areas of the external ear and in the opening of the external acoustic meatus. The LRCs in the opening of the external acoustic meatus was much higher than that of the distal and middle areas (N = 6).

To determine the distribution of LRCs in the auricular cartilage, the auricles of 24-week-old BrdU-labeled mice were analyzed (Figure 4A). Interestingly, the proximal part, i.e., the opening of the external acoustic meatus, contained a higher percentage of LRCs (23.87±0.07%) than the distal (0.02±0.03%) or middle (0.01±0.00%) parts of the auricle (N = 3) (Figure 4B).

### Specific expressions of CD44 and integrin α_5_ in auricular perichondrium, but not chondrium

To further characterize the LRCs (putative stem cells), the auricles of 24-week-old mice were stained with antibodies against well-characterized cell-surface molecules. Integrin-α_1_, α_2_, α_L_, and α_V_ were expressed by all cells in the chondrium (Figure S3). Cells in the perichondrium and the chondrium expressed integrin-β_1_, but not integrin-α_6_, α_M_, α_X_, β_2_, β_3_, syndecan-1, 3, 4, PECAM, VCAM-1, or Flk-1. Interestingly, expression of CD44 and integrin-α_5_ was specific to the perichondrium, indicating that these molecules may be markers for the putative auricular stem/progenitor cell population (Table 1).

**Table 1:** Cell surface marker characterization of chondrium and perichondirum layer using 24-week-old mice auricles.

Marker	Perichondrium	Chondrium
Integrin alpha-1	−	++
Integrin alpha-2	−	++
Integrin alpha-5	++	−
Integrin alpha-6	−	−
Integrin alpha-V	−	++
Integrin alpha-L	−	++
Integrin alpha-M	−	−
Integrin alpha-X	−	−
Integrin beta-1	++	++
Integrin beta-2	−	−
Integrin beta-3	−	−
Syndecan 1	−	−
Syndecan 3	−	−
Syndecan 4	−	−
PECAM^a^	−	−
VCAM 1^b^	−	−
Flk 1^c^	−	−
CD44	+	−

NOTE. (−) = no immunostained cells; (+) = 0<positive cells ≤5%; (++) = over 5% cells positive.

a ; PECAM 1 = platelet/endothelial cell adhesion molecule.

b ; VCAM 1 = vascular cell adhesion molecule 1.

c ; Flk1 = Fetal Liver Kinase1.

### Characterization of long-term LRCs in vitro

**Figure 5:**
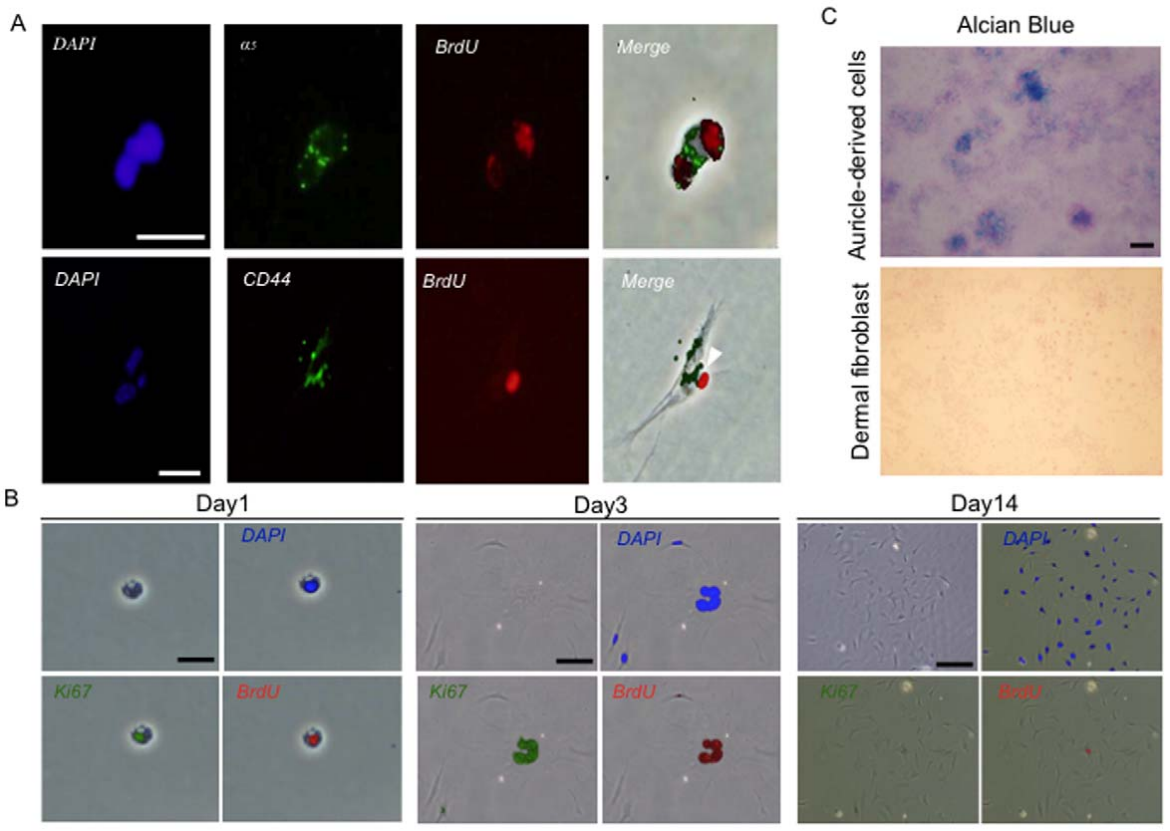
In vitro characterization of CD44+ integrin-α5+ LRCs. (A) Immunocytochemistry of LRCs in vitro. Integrin-α5 and CD44 were co-expressed in BrdU-labeled cells. CD44 staining: scale bar = 20 µm; integrin-α5 staining: scale bar = 40 µm. Original magnification: ×200. (B) Colony formation of LRCs. Cells from 24-week-old mice, which were injected with BrdU as E17 to E19 fetuses, were harvested from mice auricle following collagenase digestion. Colony assay was performed to examine the clonogenicity of LRCs. Cells were stained at 1, 3 and 14 day after plated. Clonal colonies were stained with antibodies against DAPI, Ki-67 and BrdU. Scale bars = 20 µm or 100 µm (C) Alcian blue staining of cells isolated from auricular cartilage or dermal fibroblasts. Scale bar = 200 µm.

Based on the immunohistochemistry analyses, we next examined whether auricular LRCs expressed CD44 and integrin-α_5_. Long-term BrdU labeled mice auricles were harvested at 24 weeks post birth. After enzymatic digestion of harvested auricle, we successfully isolated and cultivated the LRCs *in vitro*. Primary cultures of auricle cells isolated from 24-week-old BrdU-labeled mice were stained with antibodies against CD44 and integrin-α_5_. The long-term LRCs expressed both CD44 and integrin-α_5_, indicating both are potential stem cell markers (Figure 5A).

To examine the clonogenic capability of LRCs in BrdU-labeled 24-week-old mice auricle, cells were plated onto a cell culture dish under clonal culture condition (1,000 cells/cm^2^). Immunocytochemistry showed that long-term LRC possessed a capacity to form a colony following 14 days cultivation (Figure 5B). Interestingly, BrdU-label was confined to a part of cells inside the colonies. This phenomenon seemed to be a result of asymmetric DNA segregation, indicating an asymmetric cell division, that is, one major characteristic of stem cells [16].

Identification of stem/progenitor cells will facilitate future efforts towards auricular cartilage regeneration. To determine the chondrogenic potential of cells, semi-clonally expanded cells containing long-term LRCs were cultivated under high-density condition in vitro. Alcian blue staining showed the production of cartilage proteoglycans, indicating the chondrogenic potential (Figure 5C).

## Discussion

We demonstrated the presence of putative stem/progenitor cells in auricular elastic cartilage using a BrdU-labeling assay, a well-established technique that identifies stem cells based on their quiescent nature [17]. Long-term LRCs were found in the perichondrium, surrounding the auricular chondrium. LRCs are characterized by having a longer cell cycle than actively proliferating cells. During cartilage growth and maturation, mature cells experience a transient period of active proliferation and finally execute a terminal differentiation program. As a result, BrdU labeling diminishes during several cycles of division in the active proliferation period. We successfully incorporated the BrdU label into the majority of the cells of E17 fetuses that were to develop into the auricle. Observations the 48 weeks after the birth of BrdU-labeled offspring revealed that long-term LRCs remained at a rate of 0.08±0.06% in perichondrium and that no cells in the chondrium retained BrdU label. Consistent with this, Ki67 immunocytochemistry showed that during the 4 weeks following birth, cells in chondrium were proliferating vigorously and contributed to the rapid enlargement of auricle. However, after 4 weeks, no proliferating cells were detected in the chondrium. In contrast, one week after birth, no proliferating cells were found in the perichondrium. Stem/progenitor cells go into a dormant state more rapidly than differentiated cells; therefore, the long-term LRCs in the perichondrium may be a putative stem/progenitor population in auricular cartilage. Furthermore, this population of rare slow-cycling cells may contribute to long-term maintenance of the auricle.

Several reports indicate that stem cells are present in hyaline cartilage. The surface layer, growth plate, and synovium of the articular cartilage contain multipotent stem/progenitor cells [18], [19], [20]. The fibrous layer surrounding costal cartilage contains cells with regenerative capacity, and the cartilage surrounding costal bone might have high growth activity [21]. However, there are no reports that describe the stem/progenitor cells in the elastic cartilage of mice. Using a long-term LRC assay, we demonstrated that a population of putative stem/progenitor cells was present in the auricular perichondrium of 48-week-old mice. Interestingly, most LRCs were in the opening of the external acoustic meatus, but not at the distal part of the auricle. Generally, injured auricular cartilage has a limited capacity to heal, resulting in uncontrollable scar formation. Further characterization of stem/progenitor cells may help to develop a method to guide these cells to differentiate and treatments that can control scar formation. Finally, maximizing intrinsic regenerative capacity may lead to a novel strategy for auricular regeneration or repair.

Thus, characterization of LRCs, i.e., stem cells, will pave the way to for cartilage regenerative therapies. Based on the cell-surface markers expressed in articular chondrocytes, we analyzed expression of several cell-surface molecules to identify markers for the putative stem cell population in the auricular perichondrium. Of the molecules analyzed, CD44 and integrin-α_5_ antigens were specifically expressed in cells of the auricular perichondrium. CD44 is a multifunctional adhesion molecule that binds to hyaluronan (HA), type I collagen, and fibronectin. Mesenchymal stem cells, neural crest-derived cells, and embryonic stem cells express CD44 antigens [22], [23], [24], [25]. Integrin-α_5_, a type 1 collagen and fibronectin ligand, is a component of the auricular perichondrium [26], [27], [28], [29], [30], [31]. Stem cells in the articular surface and growth plate also express integrin-α_5_, indicating that these are potential stem/progenitor cell markers [26], [32], [33]. Based on these observations, long-term LRCs were examined in vitro and shown to co-express CD44 and integrin-α_5_. Both molecules are thus promising markers for future enrichment of stem cells.

In conclusion, we identified long-term label-retaining stem/progenitor cells in auricular perichondrium by analyzing BrdU-labeled auricles of mice. Utilization of these stem/progenitor cells from auricular cartilage will not only improve our understanding of basic cartilage biology, but it may also lead to novel therapeutic strategies, including long-term tissue restoration, for patients with craniofacial defects.

## Materials and Methods

### Time-dependent changes in the surface area of the external ear and in the thickness of auricular cartilage

The surface area of the external ear was calculated using measurements from the bottom of the ear lobe to the distal end of the auricle. The thickness of the auricular cartilages was measured in Alcian blue-stained specimens using 4 equidistant points from the bottom of the ear lobe to the distal end of the auricle. The surface area and thickness were measured on days 1, 2, and 4 and weeks 24 and 48 after birth. Mean values were calculated from the measurements of 6 mice using the WinROOF software (MITANI, Fukui, Japan).

### BrdU labeling

To label slow-cycling cells, ICR pregnant mice with embryonic day 17 mice (Japan SLC Co, Shizuoka, Japan) were injected with BrdU (50 µg/g body weight [WTg]) (Sigma-Aldrich, St. Louis , MO) intraperitoneally on twice daily for 3 days. The offspring were sacrificed as newborns or on day 2, day 4, week 24, or week 48 following birth. All animal experimentation was conducted in accordance with the Guidelines for Proper Conduct of Animal Experiments (Science Council of Japan), and all experimental procedures were approved by institutional review board of Animal Research Center, Yokohama City University School of Medicine (11–68).

### Histochemical and immunohistochemical analysis

Tissue sections were stained with Hematoxylin/eosin or Alcian blue. For immunohistochemical analysis, the tissue sections were immunolabeled with primary antibodies: anti-BrdU antibody (sheep, 1∶1200; Sigma-Aldrich, St. Louis, MO, USA) Ki67 (rabbit, 1∶200; Abcam, Cambridge, MA, USA) α1-integrin (hamster, 1∶100; BD Biosciences, San Jose, CA USA), α2-integrin (hamster, 1∶100; BD Biosciences), α5-integrin (rabbit, 1∶100; Chemicon, Temecula, CA USA), α6-integrin (rat, 1∶100; Chemicon), αV-integrin (rabbit, 1∶100; Chemicon), αL-integrin (rat, 1∶100; eBioscience, San Diego, CA, USA), αM-integrin antibody (rat, 1∶100; eBioscience), αX-integrin (hamster, 1∶100; eBioscience), β1-integrin (hamster, 1∶100; BD Biosciences), β2-integrin (rat, 1∶100; eBioscience), β3-integrin (hamster, 1∶100; BD Biosciences), CD44 (rat, 1∶100; Chemicon), Syndecan-1 (rat, 1∶100; BD Biosciences), Syndecan-3 (rabbit, 1∶100; Santa Cruz Biotechnology, Santa Cruz, CA, USA), Syndecan-4 (rat, 1∶100; BD Biosciences), PECAM (rat, 1∶100; BD Biosciences), VCAM-1 (rat, 1∶100; Chemicon), or Flk-1 (rat, 1∶100; BD Biosciences) at 4°C overnight.

After washing, the sections were incubated with secondary antibody, Cy3-conjugated Donkey anti-sheep IgG antibody (1∶1600; Chemicon), Cy3-conjugated Donkey anti-Rabbit IgG antibody (1∶1600; Jackson Immuno Research Laboratories, Inc. West Grove, PA, USA), and/or Alexa488-conjugated Donkey anti-rabbit IgG antibody (1∶1200; Molecular Probes, Junction City, OR, USA) depending on the primary antibody used. Tissue sections were incubated with secondary antibody for 1 h at room temperature. The samples were counterstained with DAPI and analyzed with a LSM510 laser-scanning microscope (Zeiss).

### Calculation of BrdU-labeling Index (LI) and the Ki67-positive cell Index (KI)

The number of BrdU-positive cells was divided by the total number of DAPI-stained cells in a field to calculate the residual LRC percentage within a field of cells. The mean LRC percentages from five fields at ×100 magnification were calculated to determine the BrdU-labeling Index (LI) for each time point. To compare the rate of cell division, the Ki67-positive cell Index (KI) was calculated from the mean of the Ki67 ratio in five fields at ×100 magnification. The LI and KI were calculated separately for the auricular perichondrium and the chondrium at days 0, 2, and 4, and weeks 24 and 48 following birth.

### Isolation and cultivation of mice auricular cartilage

Auricular cartilage was obtained from 24-week-old, BrdU-labeled mice under a SZ 60 Stereo Microscope (Olympus). Dissected tissues were cut into small pieces and digested in Hank's balanced solution (Sigma) with 0.2% collagenase type II (Worthington) during a 2-h incubation at 37°C with shaking.

After passing the tissue suspension through a 40-µm nylon mesh (BD Falcon), the cells were washed three times with PBS. The isolated cell suspensions were cultured in Dulbecco's modified Eagle medium and Ham's F-12 medium (D-MEM/F-12; Nissui Pharmaceutical Japan) supplemented with 10% fetal bovine serum (FBS; Moregate, Bulimba city, Australia and 1% antibiotic antimycotic solution (AMS; Sigma) in 5% CO_2_ at 37°C. Cells were seeded in 10%FBS+DMEM at the density of 10^2^/cm^2^.

## Supporting Information

Figure S1 **BrdU labeling of auricular cartilage in a 4-week-old mouse.** 4-week-old mice were injected with BrdU and were sacrificed the day. None of BrdU-labeled cells and Ki67-positive cells were observed in both the perichondrium and chondrium of an auricular cartilage. From the left, Alcian blue staining, DAPI, BrdU, Ki67, and a merged image. Two-headed arrows: the cartilage width including perichondrium. Scale bar = 100 µm.
(TIFF)

Figure S2 **Long-term LRCs specifically reside in auricular perichondrium.** 24 (A) or 48 (B) weeks-old mice auricle were immunohistochemically examined. Although none of BrdU labering cells was recognized in chondrium, rare long-term LRCs specifically existed in perichondrium layer. Two-headed arrows: perichondrium width, but not chondrium of auricular cartilage. Arrowheads: long-term LRCs. Scale bars = 200 µm.
(TIFF)

Figure S3 **Immunohistochemical analysis of cell surface marker proteins in a 24-week-old mouse.** Perichondrocytes of 24-week-old mice expressed integrin-α5 and CD44 (Arrowheads). Chondrocytes expressed integrin-α1,2,V,L and integrin-β1. Two-headed arrows: the cartilage width including perichondrium. Scale bar = 50 µm.
(TIFF)
