# Supplementary figures and images for: Presence of Cartilage Stem/Progenitor Cells in Adult Mice Auricular Perichondrium
Source: PLoS One. 2011 Oct 19;6(10):e26393. doi: 10.1371/journal.pone.0026393 (PMC3198405; doi:10.1371/journal.pone.0026393)

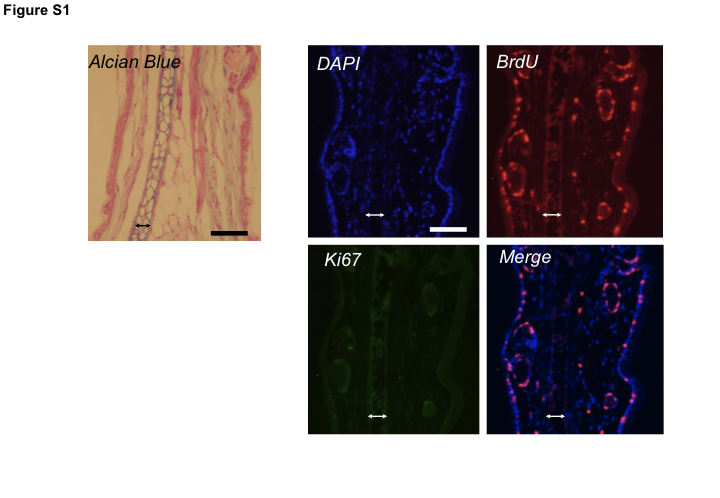

Supplement: Figure S1 — BrdU labeling of auricular cartilage in a 4-week-old mouse. 4-week-old mice were injected with BrdU and were sacrificed the day. None of BrdU-labeled cells and Ki67-positive cells were observed in both the perichondrium and chondrium of an auricular cartilage. From the left, Alcian blue staining, DAPI, BrdU, Ki67, and a merged image. Two-headed arrows: the cartilage width including perichondrium. Scale bar = 100 µm. (TIFF) [file pone.0026393.s001.tiff]

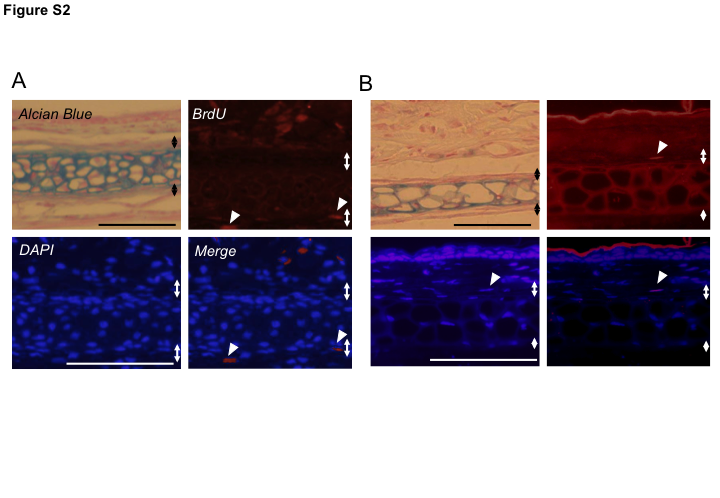

Supplement: Figure S2 — Long-term LRCs specifically reside in auricular perichondrium. 24 (A) or 48 (B) weeks-old mice auricle were immunohistochemically examined. Although none of BrdU labering cells was recognized in chondrium, rare long-term LRCs specifically existed in perichondrium layer. Two-headed arrows: perichondrium width, but not chondrium of auricular cartilage. Arrowheads: long-term LRCs. Scale bars = 200 µm. (TIFF) [file pone.0026393.s002.tiff]

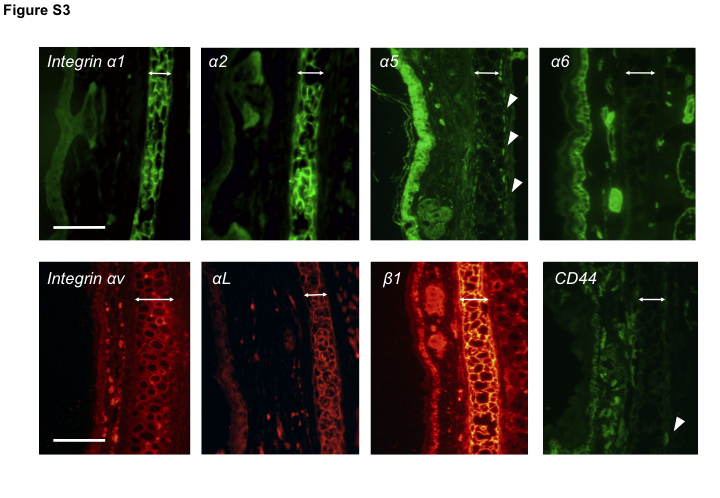

Supplement: Figure S3 — Immunohistochemical analysis of cell surface marker proteins in a 24-week-old mouse. Perichondrocytes of 24-week-old mice expressed integrin-α5 and CD44 (Arrowheads). Chondrocytes expressed integrin-α1,2,V,L and integrin-β1. Two-headed arrows: the cartilage width including perichondrium. Scale bar = 50 µm. (TIFF) [file pone.0026393.s003.tiff]
